# Norbert Donner-Banzhoff: Die ärztliche Diagnose: Erfahrung – Evidenz – Ritual

**DOI:** 10.3205/zma001596

**Published:** 2023-04-17

**Authors:** Sigrid Harendza

**Affiliations:** 1 Universitätsklinikum Hamburg-Eppendorf, III. Medizinische Klinik, Hamburg, Germany

## Bibliographical details

Norbert Donner-Banzhoff

**Die ärztliche Diagnose: Erfahrung – Evidenz – Ritual**

Hogrefe Verlag, Göttingen

Year of publication: 2022, 356 pages, price: € 44,95

ISBN: 978-3-45-686194-4

## Review

The most important statement of this book right at the beginning: “Diagnoses are not found, they are made”. This is likely to come as a bit of a shock to anyone who has enjoyed watching *Dr. H*ouse or is looking forward to a new episode of *Adventure Diagnosis* in the media library. However, Norbert Donner-Banzhoff manages the miracle of covering the entire spectrum of medical thought and action, including cognitive psychology and medical history perspectives, in order to explain this statement without once using the term *clinical reasoning*. Never before have I enjoyed reading about strategies for prevalence enrichment or regression to the mean so much, not to mention the excellent explanations and illustrations of the four-field table, where, after reading it, really no one can claim not to have understood it. And: the fact that tests only modify disease probabilities according to Bayes’ theorem and that the pre-test probability is decisive for this cannot be read often enough – so here also. For this is often forgotten in everyday clinical practice, as the author explains with striking and also somewhat frightening examples. Complete certainty can thus not be achieved in the always tricky contexts of diagnosing. Perhaps it would have been even more useful at these points for a better understanding of this fact not to speak of a disease being “ruled out” but rather of it being made “less likely” by a test. But this is whining on a high level.

This book is not just a guide to making a medical diagnosis, it is much more than that. It offers insight into the daily work of physicians in the trickiest task and the greatest professional challenge: making (i.e., “making”) a diagnosis. In doing so, it is devoted to historical perspectives and scientific traditions of different countries that approach the diagnostic process in different ways. This offers an excellent opportunity to reflect on one's own medical work – and teaching – and to open up to sometimes painful insights. The author discusses how reference ranges come about and that biological fluctuations are the greatest source of uncertainty, as well as the overestimation of technical findings in everyday medical practice and the harmful consequences of overdiagnosis and overtreatment. Theoretical, partly philosophical passages explaining important background information on the status quo of medical diagnosis with its problematic consequences (“X-rays and injections are powerful rituals”) alternate with current practical references. These are strikingly accessible to those working in the medical field, students and teachers, and offer good starting points for reflecting on one’s own actions.

It is very pleasant to note that, with very few exceptions, English terms have been translated into German. The chosen form of gendering – the female form is used everywhere, except when exclusively men are meant – keeps the text pleasantly readable, even if this principle weakens somewhat in some places toward the end. Each chapter ends with a focused outlook that conclusively brings what has been read to the point and offers a good summary of the essential aspects in each case. Despite its broad scientific basis, the book reads almost like a novel or detective story, because the flow of reading is not disturbed by footnotes. An overview of the underlying literature can be found at the end of the chapters and, to an even greater extent, in annotated digital supplementary material that can be downloaded from a website.

The professional primary socialization is based on scientific knowledge (“disease”), whereas in daily work with patients each person represents an individual case (“illness”). From my point of view, this is the most important insight in diagnosing pointed out by Norbert Donner-Banzhoff. Each patient must be “understood” individually in order to “make” a diagnosis. This is also seen as the greatest challenge for medical teaching, which has so far paid little attention to this aspect. The complexity of medical diagnosis cannot be fully grasped by textbooks and guidelines, as is impressively described. The book leaves no doubt that it is possible to master the task of diagnosing despite all the uncertainty, despite the reservations that may arise among readers. The prerequisite for this is to critically reflect on one’s own medical actions and teaching and then to put them into practice with new impulses.

It is therefore to be hoped that this book will be read and reflected upon by many people involved in medical training, in order to adapt the undergraduate studies and thus the socialization of the next generation of doctors accordingly. The book even has a separate chapter on how this could be done. Finally, a “trigger warning”: as already mentioned at the beginning, the book contains surprising, scientifically sound insights for all those who have so far only reflectively dealt with the process of medical diagnosis to a limited extent. However, once the initial shock of what errors and problems can occur has been overcome, the first step has already been taken to better design medical training in the future.

## Competing interests

The author received a free copy of this book for this review.

